# Neighbourhood and family correlates of immigrant children’s mental health: a population-based cross-sectional study in Canada

**DOI:** 10.1186/s12888-022-04096-7

**Published:** 2022-07-05

**Authors:** Amanda Sim, Katholiki Georgiades

**Affiliations:** Department of Psychiatry and Behavioural Neurosciences, McMaster University, The Offord Centre for Child Studies, Hamilton, ON Canada

**Keywords:** Immigrant, Children, Mental health, Parenting, Family, Neighbourhood

## Abstract

**Background:**

Immigrant children exhibit significant variation in their mental health outcomes despite disproportionate exposure to socio-economic adversity compared to their non-immigrant peers. Identifying aspects of neighbourhood and family contexts that are most salient for immigrant children’s mental health can help to inform and target interventions to prevent mental disorder and promote mental well-being among this population.

**Methods:**

The study analyzed multi-informant data from 943 first- and second-generation immigrant caregiver and child dyads from the Hamilton Youth Study, a representative sample of immigrant and non-immigrant families in Hamilton, Ontario. Multivariate multilevel regression models examined associations between neighbourhood and family characteristics and processes, and parent and child self reports of internalizing and externalizing problems.

**Results:**

Positive and negative parenting behaviours were significantly associated with internalizing and externalizing problems, with negative parenting demonstrating associations with externalizing problems across both parent and child reports (*b* = 0.26–1.27). Neighbourhood social disorder and parental trauma exposure were associated with greater internalizing and externalizing problems, and neighbourhood immigrant concentration was associated with fewer externalizing problems for parent reports only. Adding parental distress and parenting behaviour to the models reduced the coefficients for parental trauma exposure by 37.2% for internalizing problems and 32.5% for externalizing problems and rendered the association with neighbourhood social disorder non-significant. Besides the parenting variables, there were no other significant correlates of child-reported internalizing and externalizing problems.

**Conclusions:**

Results highlight the importance of parenting behaviour and parental experiences of trauma and distress for immigrant children’s mental health. While not unique to immigrants, the primacy of these processes for immigrant children and families warrants particular attention given the heightened risk of exposure to migration-related adverse experiences that threaten parental and family well-being. To prevent or mitigate downstream effects on child mental health, it is imperative to invest in developing and testing trauma-informed and culturally responsive mental health and parenting interventions for immigrant families.

## Background

Immigrant children exhibit significant variation in their mental health outcomes despite disproportionate exposure to socio-economic adversity compared to their non-immigrant peers [1]. Identifying the factors that matter most for immigrant children’s mental health, particularly in their most proximal environments of the family and neighbourhood, can help to elucidate why some immigrant children thrive while others falter, which in turn can inform targets for interventions [2]. Such evidence has significant implications for immigrant-receiving societies such as Canada, where over a third of all children under 15 has at least one foreign‑born parent and population projections estimate that half will be of immigrant background by 2035 [3]. Compared to non-immigrants, recent immigrant families in Canada are more likely to live below the poverty line, have lower mean levels of household income, and live in neighbourhoods characterized by greater socio-economic disadvantage – all established risk factors for poor child mental health outcomes [4–6]. Immigrant families, including those who resettled as refugees fleeing persecution or armed conflict in their home countries, are also likely to experience adverse life events (e.g., trauma, discrimination) and acculturative stressors (e.g., language barriers) that can have direct and indirect effects on children’s mental health via impaired family processes [7].

This study uses data from a diverse population-based sample of first- (foreign-born) and second-generation (born in Canada to at least one foreign-born parent) immigrant children in Canada to examine associations between immigrant children’s mental health and characteristics of their neighbourhoods and families. Identifying aspects of neighbourhood and family contexts that are most salient for immigrant children’s mental health is crucial for informing and targeting interventions to prevent mental disorder and promote mental well-being among this population.

### Neighbourhood and family influences on immigrant children’s mental health

Recent conceptualizations of immigrant children’s adaptation and psychological adjustment adopt a multilevel approach integrating risks and resources at the global, political-social, microsystem, and individual-level contexts [8]. Of these, microsystems such as neighbourhoods and families are understood to be proximal contexts for all children, including immigrant children, constituting profound and enduring influences on their mental health [9].

Neighbourhood disadvantage, as assessed by socio-economic indicators (e.g., poverty, unemployment rates) and social processes (e.g., neighbourhood social disorder), has been linked to adverse child mental health outcomes [5]. These effects may occur through several pathways, including poorer availability or quality of health and social services in the community, as well as lack of collective efficacy around children’s safety and well-being [10]. In addition, studies have shown that living in neighbourhoods characterized by high levels of disorder (e.g., drug and alcohol use, crime) may increase risk of adverse childhood experiences such as maltreatment and family dysfunction, which in turn may be associated with higher levels of child mental health problems [11]. While immigrant families tend to reside in less affluent and more unstable neighbourhoods [4], evidence on the extent to which associations between neighbourhood disadvantage and poor mental health outcomes hold for immigrant children is mixed. Research with Latino immigrant youth in the United States has found both positive and null associations between neighbourhood poverty and internalizing problems [12, 13], and there appears to be little evidence of direct or indirect effects of individual perceptions of neighbourhood safety and cohesiveness on immigrant and refugee children’s mental health [14, 15].

There is more consistent support in the literature for neighbourhood immigrant concentration as a potential protective resource, with several studies demonstrating associations between higher immigrant concentration and more positive mental health outcomes for immigrant children and youth [4, 12]. The protective effect of immigrant concentration may be explained by the concept of person-environment fit [16], which posits that similarities in socio-economic, cultural, and ethnic characteristics may enhance opportunities for social cohesion, collective efficacy, and shared norms and values that buffer immigrant children from poor mental health outcomes [17].

Neighbourhood influences on immigrant children’s mental health may also operate indirectly through family processes, for example, by reducing families’ access to financial, social, and institutional resources and increasing family stress and dysfunction [10, 18]. The Family Stress Model posits that economic stressors increase the risk of child mental health problems through parental distress and impaired parenting [19, 20]. This model could be extended to include other types of stressors such as parental exposure to traumatic or adverse life experiences such as discrimination [21], particularly in view of growing evidence showing intergenerational links between parental trauma and distress, impaired parenting, and child psychopathology in the general population and in refugee and immigrant populations [22, 23].

Many immigrant families also occupy intersecting social positions (e.g., low-income, refugee status, low educational attainment) that may increase risk of poor mental health among their children [24]. There is a large body of evidence linking family poverty and its correlates to a range of negative outcomes for children. Studies have demonstrated both direct and mediated effects of family poverty via parental and child stress, family conflict, and access to resources [25]. These patterns, however, appear to be more nuanced among immigrant groups. Analysis of the Canadian National Longitudinal Survey of Children and Youth (NLSCY) found that the association between family poverty and externalizing problems was attenuated among children living in recent immigrant families compared to non-immigrant children [4, 26]. Other universal and migration-specific socio-demographic factors such as parental educational attainment, refugee status, and proficiency in the host country language, have demonstrated null or inconsistent associations with child mental health in a non-probability-based sample of Canadian immigrant and refugee youth across six major urban areas [27].

### Inter-informant variation in mental health correlates

The ability to draw conclusions from existing research on children’s mental health is further challenged by discrepancies in parent’s and children’s ratings of mental health and mental health correlates. Inter-informant discrepancy in child mental health ratings is a well-established phenomenon that may be more pronounced among immigrant and ethnic minority groups due to cultural and intergenerational differences in appraisals and expectations of child behaviour [28, 29]. Yet few studies have systematically examined inter-informant variation in correlates of immigrant children’s mental health, despite evidence of predominantly informant-specific associations in the general population [28]. Reliance on single informant reports in studies on immigrant children’s mental health poses the risk of important correlates being unrecognized or misidentified, which in turn has implications for the development and targeting of interventions [30].

### The current study

The current study aims to address gaps in the extant literature by examining associations between immigrant children’s mental health and universal and migration-specific risk and protective factors in the neighbourhood and family contexts. These risk and protective factors are clustered around five interrelated domains from the most distal to the most proximal: neighbourhood structural characteristics (i.e., immigrant concentration, median household income, unemployment rate, neighbourhood social disorder); family socio-demographic characteristics (i.e., parental refugee status, recency of arrival in Canada, English language proficiency, educational attainment, low-income); parental exposure to trauma and discrimination; parental distress; and positive and negative parenting behaviour. Specific study objectives were to: (1) examine associations between characteristics of neighbourhood and family contexts and immigrant children’s internalizing and externalizing problems; 2) assess the extent to which the magnitude of associations with more distal neighbourhood and family correlates are reduced by taking into account more proximal parental distress and parenting variables; and 3) examine differences in correlates of immigrant children’s mental health across parent versus child self reports of internalizing and externalizing problems.

## Methods

### Participants and procedure

We analyzed data from the Hamilton Youth Study (HYS), a probability-based cross-sectional survey of 1,449 first-generation immigrant (born outside Canada), second-generation immigrant (born in Canada to at least one parent born outside of Canada), and non-immigrant (born in Canada to Canadian-born parents) children in grades 5–8 attending 36 elementary schools in Hamilton, Ontario. Hamilton has a large and diverse immigrant population, ranking fifth nationally and second in Ontario for its proportion of foreign-born population at 24.1% [31]. For the purposes of this study, only first and second-generation immigrant children were included in the sample (*n* = 943).

Given the comparative nature of the HYS, cost-effective sampling methods were developed to enlist a representative sample of immigrant students and a comparison of non-immigrant students living in similar neighbourhoods and attending the same schools. Assuming a response rate of 75% at the student level and a sampling strategy designed to enlist equal numbers of immigrant (first and second generation) and non-immigrant students per school (12/group), schools with a minimum of 16 first-generation immigrant students were eligible to participate (*n* = 54 schools). A two-stage (school, student), stratified random sampling method was used to select schools and students. At stage 1, schools were stratified by percentage of first-generation immigrant students (low, medium, high) and a simple, random sample of 12 schools was selected from each stratum. School sampling weights were generated on the basis of the probability of selection (1/probability of selection). At stage 2, students within schools were stratified on the basis of country of birth (i.e., in or outside Canada) and first language (i.e., English/not English), and simple random samples from each stratum were selected for a total of 36 students per school (i.e., 12 students born outside of Canada, 12 students born in Canada but whose first language was not English and 12 students born in Canada whose first language was English). Student level sampling weights were generated on the basis of the probability of selection (1/probability of selection).

A letter was sent home to selected families introducing the study and inviting their participation. Families who did not wish to be contacted further about the study were asked to indicate this on the letter and return it to the school. All other families were contacted by telephone by research staff to introduce the study, obtain informed consent and schedule an interview. Written parental consent and child assent were obtained prior to conducting separate interviews with the child and the person most knowledgeable about the child, usually the parent. In order to address language-related barriers to participation, interviews were conducted in the respondent’s home or at the child’s school by a trained multilingual team of interviewers using study materials translated into 8 languages (Arabic, Urdu, Spanish, Vietnamese, Serbian, Chinese, Tagalog and Somali) in addition to English. Response rates were 92% at the school level and 70% at the student level. The study was approved by the Hamilton Integrated Research Ethics Board.

### Measures

#### Neighbourhood characteristics

Neighbourhood was defined as the dissemination area (DA) used in the 2011 National Household Survey (NHS), comprising a population of 400 to 700 persons [32]. Data for neighbourhood variables were derived from the 2011 NHS using participants’ postal code to link to the DA. Neighborhood-level variables derived from the 2011 NHS at the DA level included: percentage of first-generation immigrants, median family income, and unemployment rate. To obtain a measure of neighbourhood social disorder, parents’ responses to 7 items (e.g. *How much of a problem are litter, broken glass or garbage in your neighbourhood?*) adapted from the Project on Human Development in Chicago Neighborhoods Community Survey [33] were summed and scores were aggregated up to the DA level by estimating a mean score across all parent responses within a given DA.

#### Family demographic characteristics

These variables included: (1) refugee status, assessed by the parent’s response to the item “Did you come to Canada as a refugee?” (0 = *no*, 1 = *yes*); (2) number of years since arrival in Canada; (3) parental educational attainment (0 = *Bachelor’s or above*, 1 = *below Bachelor’s*, 2 = *high school or less*); (4) parents’ self-rated English proficiency (1 = *poor*, 2 = *proficient*, 3 = *fluent/native*); and (5) household poverty derived from parental self-reports of total household income and using the 2010 Statistics Canada low-income measure (LIM) (0 = *not low income*, 1 = *low income*) [34].

#### Parental exposure to trauma and discrimination

Lifetime trauma exposure was assessed by 12 items (e.g. “*Have you ever been hit, punched or kicked very hard?*”) adapted from Part 1 of the Harvard Trauma Questionnaire (HTQ), a self-report measure developed by expert consensus methods to measure experience of trauma [35]. The HTQ has been widely translated and used with refugee and other trauma-exposed clinical and community samples around the world and has shown excellent temporal stability and internal consistency [36]. Responses to the 12 items used in our study were summed to reflect the number of different traumatic events experienced by participants, given evidence of a dose–response effect of trauma exposure on depression and post-traumatic stress symptoms [37].

Perceived discrimination was assessed by the Experiences of Discrimination (EOD) Scale, which is composed of 9 items assessing frequency of perceived discrimination due to the participant’s race or ethnicity in a variety of situations in the past 12 months (e.g. *Getting hired or getting a job*) [38]. The EOD scale was found to be a valid and reliable self-report measure of discrimination in an ethnically diverse community sample, demonstrating adequate internal consistency and test–retest reliability and good construct validity [38]. Internal consistency reliability in this sample was 0.73. Responses (0 = *never*, 1 = *once*, 2 = *2–3 times*, and 3 = *4 or more times*) were summed to obtain an overall score of perceived discrimination (min = 0, max = 27), which was used in the analysis as a categorical variable consisting of high (4–27), moderate/low (1–3), and no discrimination (0).

*Parental distress* was assessed by the 10-item Kessler Psychological Distress scale (K-10) [39]. The K-10 is widely used to screen for anxiety and depression and previous studies with culturally and ethnically diverse samples have shown evidence of a single factor structure and good internal consistency reliability and criterion validity [40]. Parents were asked how frequently they experienced symptoms of nonspecific psychological distress during the past 30 days (e.g., *Feeling so depressed that nothing could cheer you up*). Responses (1 = *none of the time*, 2 = *a little of the time*, 3 = *some of the time*, 4 = *most of the time*, 5 = *all of the time*) were summed to obtain an overall score of parental distress (min = 10, max = 50). Internal consistency reliability in this sample was 0.89.

#### Parenting behaviour

Parents and children responded to 18 items on parenting behaviours drawn from the Parent Behavior Inventory [41] and Cycle 3 of the NLSCY [42] (e.g., *I smile at* < *child name* > *very often*, *I threaten to hurt* < *child name* >). Exploratory factor analysis with Promax rotation showed that 10 items describing positive parenting behaviours loaded onto one factor, while 5 items describing negative parenting behaviours loaded onto a second factor. The remaining 3 items did not load onto either factor and were dropped from the analysis. Responses (1 = *never*, 2 = *rarely*, 3 = *sometimes*, 4 = *often*, and 5 = *always)* were summed to obtain parent and child scores of positive (min = 10, max = 50) and negative (min = 5, max = 25) parenting. Internal consistency reliability was 0.89 and 0.64 for parent-reported positive and negative parenting respectively, and 0.92 and 0.69 for child-reported positive and negative parenting respectively. Correlations between parent- and child-reported positive (*r* = 0.22, *p* < *0.001*) and negative parenting behaviours (*r* = 0.12, *p* < *0.001*) were small in magnitude.

#### Child internalizing and externalizing problems

We used 104 items from the Child Behavior Checklist for Ages 6–18 (CBCL/6–18) and Youth Self-Report (YSR) to assess internalizing and externalizing problems in the past 6 months [43]. The CBCL and YSR have been translated into over 100 languages and validated with diverse cultural groups, with findings from 44 societies in Asia, Africa, Europe, South America, and North America showing international consistency in model fit and age and gender patterns [44]. Raw scores were converted into standardized *t*-scores for the broadband scales of internalizing (withdrawn, somatic complaints, and anxious/depressed subscales) and externalizing (delinquency and aggression sub-scales) behaviours. Correlations between parent- and child-reported internalizing (*r* = 0.16, *p* < 0.001) and externalizing problems (*r* = 0.20, *p* < 0.001) were small in magnitude, consistent with meta-analytic reviews showing low correspondence between informants’ reports on child mental health [28].

### Analyses

Data were analyzed using multivariate multilevel regression given the hierarchical structure of the data with children (Level 1) nested within schools (Level 2). Sampling weights were applied at the student and school levels based on the probability of selection. The multivariate regression models were estimated using full information maximum likelihood (FIML) with robust standard errors in Mplus version 7.11 [45]. All continuous predictor variables were grand-mean centred. To address research objective 1, we constructed a series of models to examine associations between neighbourhood and family level correlates and child mental health. Model 1 included neighbourhood structural characteristics (immigrant concentration, median income, unemployment, and neighbourhood social disorder). Model 2 added family socio-demographic characteristics (parental refugee status, recency, English language proficiency, educational attainment, and household poverty). Model 3 added parental lifetime trauma exposure and perceived discrimination. Models 4 and 5 added parental distress and positive and negative parenting respectively. We controlled the False Discovery Rate (FDR) to account for multiple hypothesis testing. The FDR is the expected fraction of tests declared to be statistically significant when the null hypothesis is actually true [46]. The FDR approach sets a threshold for the *q*-value (typically 0.05) that specifies how many false positives are acceptable among all tests declared to be significant. *P*-values for each test were calculated and rank ordered in SPSS and those at or below the pre-determined *q*-value of 0.05 were deemed to be statistically significant for the purposes of our study.

To address research objective 2, we examined the change in coefficients associated with neighbourhood and family characteristics and parental exposure to trauma and discrimination after the addition of parental distress and parenting variables in Models 4 and 5. Finally, to address research objective 3 we ran Wald chi-square tests of differences in coefficients to compare the strength of association between each statistically significant correlate and parent versus child self-reports of internalizing and externalizing behaviors in Model 5. All models controlled for the age, sex (male), and 1^st^ generation immigrant status of the child.

## Results

Table 1 presents sample characteristics. Over half of the children in the sample (59.2%) were first-generation immigrants and over a quarter (28.2%) of families arrived in Canada as refugees. The majority (57.1%) of immigrant families in the sample were low income and a quarter of parents had a high school education or less. Tables 2 and 3 present the multivariate response models of parent and child-reported internalizing and externalizing problems respectively. We discuss below only the estimates that were significant after controlling for the FDR.

**Table 1:** Sample characteristics (*n* = 943)

*Characteristic*				*Min, Max*^*a*^
*Child*				
Age, *M* (*SD*)		12.22 (1.23)		9, 15
Male (%)		47.5		-
1st generation (%)		59.2		-
Externalizing problems—parent, *M* (*SD)*		44.45 (8.67)		33, 77
Externalizing problems—child, M (SD)		44.83 (9.79)		29, 84
Internalizing problems—parent, M (SD)		48.41 (10.82)		33, 84
Internalizing problems—child, M (SD)		51.27 (10.07)		27, 83
*Family*				
Below Low-Income Measure (LIM) (%)	57.1		-	
Parental educational attainment (%)				
High school or less	24.6		-	
Below Bachelor's	26.6		-	
Bachelor's or above	48.8		-	
Parental English fluency (%)				
Native speaker/fluent	38.8		-	
Proficient	53.2		-	
Poor	8.0		-	
Parent arrived as refugee^b^ (%)	28.2		-	
Parent years in Canada, *M* (*SD*)	16.73 (12.33)		0, 59	
Parental lifetime trauma exposure, *M* (*SD*)	1.75 (2.16)		0, 11	
Parental perceived discrimination (%)				
High	22.4		-	
Low	25.0		-	
None	52.6		-	
Parental distress, *M* (*SD*)	16.21 (6.81)		10, 50	
Parenting behaviour				
Positive parenting—parent, *M* (*SD*)	46.22 (4.34)		29, 50	
Positive parenting—child, *M* (*SD*)	44.69 (6.01)		10, 50	
Negative parenting—parent, *M* (*SD*)	8.43 (2.81)		5, 20	
Negative parenting—child, *M* (*SD*)	8.69 (2.92)		5, 24	
*Neighbourhood*				
First generation immigrants, % (*SD*)	34.17 (14.14)			-
Median household income (in thousands), *M* (*SD*)	68.37 (27.11)			21.22, 151.60
Unemployment rate, *M* (*SD*)	9.42 (8.75)			0, 46.20
Level of neighbourhood disorder, *M* (*SD*)^c^	8.87 (1.60)			7, 19

*M* = Mean; *SD* = Standard deviation

^a^ Minimum and maximum observed values

^b^ Includes parents who arrived in Canada as a refugee, was or is currently a refugee claimant/ asylum seeker or has ever lived in a refugee camp

^c^ Parent-reported neighbourhood disorder aggregated at the Census Dissemination Area

**Table 2:** Multivariate Response Model of Parent and Child-Reported Internalizing Problems, Weighted (*n* = 943)

Characteristic	Neighbourhood (Model 1)		Family demographics (Model 2)		Adverse life experiences (Model 3)		Parent distress (Model 4)		Parenting (Model 5)	
	Parent (*b*, SE)	Child (*b*, SE)	Parent (*b*, SE)	Child (*b*, SE)	Parent (*b*, SE)	Child (*b*, SE)	Parent (*b*, SE)	Child (*b*, SE)	Parent (*b*, SE)	Child (*b*, SE)
*Fixed effects*										
Intercept	48.06 (0.70)***	49.001 (0.696)***	46.06 (1.481)***	48.007 (0.849)***	45.773 (1.609)***	48.396 (0.896)***	46.566 (1.595)***	48.315 (0.909)***	46.405 (1.544)***	49.359 (0.836)***
Neighbourhood										
% 1st generation immigrants	-0.039 (0.04)	-0.012 (0.032)	-0.042 (0.038)	-0.013 (0.03)	-0.033 (0.04)	-0.011 (0.029)	-0.032 (0.037)	-0.011 (0.029)	-0.031 (0.035)	-0.005 (0.026)
Median family income (in thousands of dollars)	-0.022 (0.02)	-0.04 (0.021)	-0.005 (0.024)	-0.032 (0.02)	-0.005 (0.024)	-0.03 (0.02)	-0.003 (0.026)	-0.03 (0.02)	-0.017 (0.023)	-0.035 (0.015)*
Unemployment rate	-0.045 (0.043)	0.133 (0.055)*	-0.062 (0.043)	0.13 (0.054)*	-0.08 (0.047)	0.124 (0.055)*	-0.06 (0.046)	0.126 (0.055)*	-0.066 (0.045)	0.11 (0.052)*^
Neighbourhood disorder^a^	0.666 (0.215)**	-0.054 (0.312)	0.663 (0.216)**	-0.08 (0.323)	0.634 (0.217)**	-0.034 (0.321)	0.57 (0.232)*	-0.042 (0.322)	0.406 (0.197)*	-0.025 (0.312)
Family socio-demographics										
Parent arrived in Canada as a refugee^b^			-0.748 (0.948)	-1.198 (1.004)	-1.84 (0.948)	-1.446 (1.033)	-1.234 (0.909)	-1.376 (1.046)	-1.097 (0.91)	-1.248 (0.989)
Years parent living in Canada			0.04 (0.08)	-0.026 (0.058)	0.045 (0.076)	-0.025 (0.056)	0.041 (0.075)	-0.026 (0.056)	0.009 (0.075)	-0.012 (0.06)
Parent English language proficiency^c^										
Poor			0.827 (1.539)	-2.644 (1.245)*	1.271 (1.421)	-2.608 (1.213)*	0.149 (1.448)	-2.642 (1.224)*	-0.177 (1.408)	-2.447 (1.26)
Proficient			0.71 (0.866)	-0.084 (0.935)	0.911 (0.931)	-0.024 (0.947)	0.385 (0.911)	-0.021 (0.953)	0.266 (0.801)	0.491 (0.732)
Parent educational attainment^d^										
High school or less			1.989 (1.05)	0.55 (0.88)	1.987 (1.005)*	0.352 (0.912)	1.652 (1.001)	0.341 (0.923)	1.748 (0.993)	0.646 (0.696)
Below Bachelor's			1.94 (1.304)	1.381 (0.908)	1.682 (1.284)	1.25 (0.911)	1.489 (1.252)	1.261 (0.922)	1.332 (1.20)	1.213 (0.781)
Household below Low Income Measure (LIM)			2.142 (1.013)*	1.941 (0.797)*	1.395 (1.013)	1.779 (0.788)*	0.394 (0.991)	1.711 (0.768)*	0.854 (0.866)	1.485 (0.71)*
Adverse life experiences										
Parental lifetime trauma exposure					1.041 (0.22)***	0.214 (0.154)	0.707 (0.228)**	0.182 (0.162)	0.654 (0.235)**^	0.186 (0.154)
Parental perceived discrimination (high)					1.37 (1.105)	-1.44 (1.177)	0.499 (1.141)	-1.465 (1.206)	0.461 (1.161)	-1.257 (0.871)
Parental perceived discrimination (low)					1.623 (0.689)*	0.809 (1.055)	1.415 (0.742)	0.838 (1.059)	1.272 (0.695)	0.406 (0.945)
Parental distress							0.435 (0.068)***	0.038 (0.053)	0.356 (0.063)***^	0.006 (0.051)
Parenting										
Positive (parent report)									-0.316 (0.127)*^	-0.039 (0.098)
Positive (child report)									-0.008 (0.062)	-0.335 (0.088)***^
Negative (parent report)									0.5 (0.196)*	0.171 (0.165)
Negative (child report)									0.071 (0.147)	1.063 (0.206)***^
Covariates										
Child age	-0.021 (0.333)	0.694 (0.319)*	-0.092 (0.327)	0.739 (0.326)*	-0.281 (0.325)	0.708 (0.312)*	-0.088 (0.336)	0.726 (0.309)*	-0.181 (0.384)	0.314 (0.283)
Child sex (male)	1.008 (0.761)	2.087 (0.949)*	1.087 (0.737)	2.196 (0.935)*	1.325 (0.678)	2.219 (0.923)*	1.781 (0.624)**	2.301 (0.914)*	1.447 (0.577)*	0.775 (0.794)
Child immigrant status (1st generation)	-0.409 (0.741)	2.023 (0.785)**	-1.331 (1.134)	1.897 (1.038)	-1.046 (1.213)	1.806 (1.029)	-0.82 (1.203)	1.883 (1.039)	-0.56 (1.254)	1.101 (0.888)
*Random effects*										
Level 2: School	3.692 (1.835)*	3.17 (1.276)*	3.685 (2.005)	2.763 (1.236)*	3.876 (2.009)	2.572 (1.119)*	3.79 (1.935)*	2.535 (1.105)*	4.088 (2.068)*	2.043 (0.924)*
Level 1: Child	101.37 (6.700)***	91.97 (5.141)***	90.589 (4.916)***	99.265 (6.785)***	93.946 (7.256)***	90.027 (4.85)***	86.976 (7.18)***	90.033 (4.882)***	81.916 (7.425)***	71.298 (4.649)***

Bolded values denote significant coefficients after controlling the False Discovery Rate (FDR) (*q*-value of 0.05). *p < .05, **p < .01, ***p < .001. ^ denotes a significant difference in parent and child-reported coefficients. All coefficients are unstandardized

^a^ Parent-reported neighbourhood disorder aggregated at the Census Dissemination Area

^b^ Includes parents who arrived in Canada as a refugee, was or is currently a refugee. claimant/asylum seeker, or has ever lived in a refugee camp

^c^ Reference group for the variable parental English language proficiency was “fluent/native”

^d^ Reference group for the variable parental educational attainment was “Bachelor’s or above”

**Table 3:** Multivariate Response Model of Parent and Child-Reported Externalizing Problems, Weighted (*n* = 943)

	Neighbourhood (Model 1)		Family demographics (Model 2)		Adverse life experiences (Model 3)		Parent distress (Model 4)		Parenting (Model 5)	
Characteristic	Parent (*b*, SE)	Child (*b*, SE)	Parent (*b*, SE)	Child (*b*, SE)	Parent (*b*, SE)	Child (*b*, SE)	Parent (*b*, SE)	Child (*b*, SE)	Parent (*b*, SE)	Child (*b*, SE)
*Fixed effects*										
Intercept	43.577 (0.514)***	43.554 (0.598)***	43.032 (1.1)***	42.81 (0.859)***	42.696 (1.105)***	43.447 (0.754)***	43.426 (1.072)***	43.532 (0.751)***	51.669 (2.197)***	60.642 (4.235)***
Neighbourhood										
% 1st generation immigrants	-0.077 (0.023)***	-0.043 (0.029)	-0.066 (0.022)**	-0.048 (0.031)	-0.058 (0.022)*	-0.044 (0.03)	-0.056 (0.021)**	-0.045 (0.029)	-0.052 (0.018)**	-0.031 (0.021)
Median family income (in thousands of dollars)	0.024 (0.018)	-0.013 (0.018)	0.036 (0.018)*	-0.009 (0.019)	0.036 (0.018)*	-0.007 (0.018)	0.037 (0.018)*	-0.007 (0.018)	0.021 (0.014)	-0.014 (0.017)
Unemployment rate	0.032 (0.032)	0.077 (0.043)	0.016 (0.029)	0.076 (0.044)	-0.001 (0.027)	0.067 (0.044)	0.014 (0.026)	0.069 (0.044)	0.005 (0.023)	0.053 (0.037)
Neighbourhood disorder^a^	0.607 (0.208)**	-0.148 (0.26)	0.575 (0.199)**	-0.161 (0.273)	0.518 (0.204)*	-0.114 (0.278)	0.484 (0.21)*	-0.111 (0.269)	0.300 (0.167)	-0.105 (0.249)
Family socio-demographics										
Parent arrived in Canada as a refugee^b^			0.091 (0.74)	0.013 (0.774)	-0.919 (0.759)	-0.439 (0.835)	-0.492 (0.687)	-0.388 (0.835)	-0.264 (0.636)	-0.283 (0.686)
Years parent living in Canada			0.094 (0.057)	-0.035 (0.072)	0.094 (0.053)	-0.042 (0.066)	0.091 (0.052)	-0.043 (0.066)	0.055 (0.050)	-0.030 (0.068)
Parental English language proficiency^c^										
Poor			-0.406 (1.172)	-0.61 (2.161)	-0.024 (1.063)	-0.71 (2.071)	-0.885 (1.08)	-0.816 (2.07)	-1.034 (0.967)	-0.404 (1.565)
Proficient			-0.18 (0.722)	0.044 (0.978)	0.02 (0.747)	0.138 (0.958)	-0.402 (0.783)	0.086 (0.943)	-0.441 (0.627)	0.717 (0.663)
Parental educational attainment^d^										
High school or less			1.897 (0.916)*	0.086 (1.103)	1.858 (0.875)*	-0.252 (1.172)	1.615 (0.877)	-0.268 (1.196)	1.861 (0.888)*	0.194 (0.895)
Below Bachelor's			1.433 (1.046)	0.476 (0.769)	1.206 (0.971)	0.274 (0.805)	1.064 (0.943)	0.288 (0.816)	0.913 (0.942)	0.22 (0.736)
Household below Low Income Measure (LIM)			1.316 (0.914)	0.657 (0.951)	0.723 (0.878)	0.527 (0.88)	-0.016 (0.849)	0.458 (0.851)	0.458 (0.777)	0.215 (0.774)
Adverse life experiences										
Parental lifetime trauma exposure					0.979 (0.138)***	0.383 (0.219)	0.75 (0.133)***	0.364 (0.217)	0.661 (0.129)***	0.330 (0.190)
Parental perceived discrimination (high)					1.27 (0.724)	-2.252 (1.225)	0.626 (0.746)	-2.311 (1.254)	0.627 (0.699)	-2.002 (1.138)
Parental perceived discrimination (low)					1.111 (0.641)	0.491 (0.902)	0.959 (0.686)	0.513 (0.907)	0.741 (0.621)	0.035 (0.710)
Parental distress							0.304 (0.054)***^	0.027 (0.052)	0.205 (0.051)***	-0.014 (0.044)
Parenting										
Positive (parent report)									-0.310 (0.087)***^	-0.024 (0.093)
Positive (child report)									-0.061 (0.043)	-0.266 (0.080)***
Negative (parent report)									0.709 (0.110)***^	0.296 (0.132)*
Negative (child report)									0.261 (0.112)*	1.271 (0.140)***^
Covariates										
Child age	-0.317 (0.252)	1.49 (0.288)***	-0.418 (0.26)	1.527 (0.284)***	-0.575 (0.253)*	1.497 (0.287)***	-0.454 (0.264)	1.493 (0.286)***	-0.595 (0.240)*	1.101 (0.292)***^
Child sex (male)	0.378 (0.679)	1.234 (0.599)*	0.461 (0.637)	1.216 (0.603)*	0.69 (0.572)	1.234 (0.582)*	0.953 (0.544)	1.242 (0.599)*	0.424 (0.449)	-0.308 (0.511)
Child immigrant status (1st generation)	1.072 (0.5)*	1.072 (0.74)	-0.654 (0.923)	1.52 (0.939)	-0.243 (0.91)	1.581 (0.984)	-0.181 (0.912)	1.554 (0.984)	0.059 (0.902)	0.824 (0.917)
*Random effects*										
Level 2: School	0.415 (1.224)	1.62 (1.73)	0.753 (1.487)	1.564 (1.811)	0.883 (1.545)	1.467 (1.772)	0.776 (1.081)	1.257 (1.553)	1.303 (0.956)	1.467 (1.367)
Level 1: Child	70.481 (3.861)***	84.422 (5.333)***	68.687 (3.325)***	84.246 (5.288)***	64.08 (3.236)***	82.849 (5.118)***	60.679 (3.153)***	83.014 (5.145)***	43.261 (1.006)***	44.424 (0.826)***

Bolded values denote significant coefficients after controlling the False Discovery Rate (FDR) (*q*-value of 0.05). *p < .05, ** *p* < .01, *** *p* < .001. ^ denotes a significant difference in parent and child-reported coefficients. All coefficients are unstandardized

^a^ Parent-reported neighbourhood disorder aggregated at the Census Dissemination Area

^b^ Includes parents who arrived in Canada as a refugee, was or is currently a refugee. claimant/asylum seeker, or has ever lived in a refugee camp

^c^ Reference group for the variable parental English language proficiency was “fluent/native”

^d^ Reference group for the variable parental educational attainment was “Bachelor’s or above

### Associations between neighborhood characteristics and parent and child-reported mental health

Among the neighbourhood variables included in Model 1, neighbourhood social disorder was associated with higher levels of parent-reported internalizing (*b* = 0.67, *p* = 0.002) and externalizing problems (*b* = 0.61, *p* = 0.004). Higher neighbourhood immigrant concentration was associated with fewer parent-reported externalizing problems (*b* = -0.08, *p* = 0.001).

### Associations between family characteristics and parent and child-reported mental health

None of the family demographic variables included in Model 2 (i.e. parental refugee status, recency, English language proficiency, educational attainment, household poverty) were associated with either parent or child-reported internalizing or externalizing problems. In Models 3 and 4, more parental trauma exposure and distress were each associated with higher levels of parent-reported internalizing (*b* = 1.04, *p* < 0.001 and *b* = 0.44, *p* < 0.001 respectively) and externalizing behaviors (*b* = 0.98, *p* < 0.001 and *b* = 0.30, *p* < 0.001 respectively). Finally, in Model 5 parent-reported positive parenting behaviour was associated with fewer parent-reported internalizing (*b* = -0.28, *p* = 0.018) and externalizing (*b* = -0.29, *p* = 0.001) problems. Parent-reported negative parenting was associated with more parent-reported internalizing problems (*b* = 0.69, *p* = 0.020), as well as higher levels of both parent and child-reported externalizing problems (*b* = 0.78, *p* = 0.001 and *b* = 0.53, *p* = 0.013 respectively). Child reports of positive and negative parenting behaviour were the only other significant correlates of child self-reported internalizing and externalizing problems. Child-reported positive parenting was associated with fewer self-reported internalizing (*b* = -0.31, *p* = 0.084) and externalizing (*b* = -0.22, *p* = 0.001) problems, while child reports of negative parenting were associated with higher levels of self-reported internalizing (*b* = 1.30, *p* < 0.001) and externalizing problems (*b* = 1.65, *p* < 0.001).

Adding the parental distress and parenting variables in Model 5 reduced the coefficients for neighbourhood disorder by 36.0% from 0.63 to 0.41 for parent-reported internalizing problems, and by 42.1% from 0.52 to 0.30 for parent-reported externalizing problems and rendered the associations non-significant. The coefficients for parental trauma exposure were also reduced by 37.2% from 1.04 to 0.65 for parent-reported internalizing problems, and by 32.5% from 0.98 to 0.66 for parent-reported externalizing problems. However, the association between parental trauma exposure and parent ratings of their children’s internalizing and externalizing problems remained significant.

### Inter-informant variation in mental health correlates

Results showed few significant correlates of child-reported internalizing and externalizing problems, with only positive and negative parenting exhibiting associations with child self-reports. As described above, neighbourhood social disorder, immigrant concentration, parental trauma, and parental distress were significant correlates of parent but not child reports of internalizing and externalizing problems. Only parent and child-reported negative parenting behaviour exhibited cross-informant associations with externalizing problems. Formal tests of differences in the strength of associations across parent and child informants revealed substantial variation in the links between family factors and parent and child mental health ratings. Parental trauma exposure, parental distress, and parent-reported positive parenting were more strongly associated with parent compared to child-reported internalizing problems, while child-reported positive and negative parenting were more strongly associated with child compared to parent-reported internalizing problems. For externalizing problems, parental distress and parent-reported positive and negative parenting were more strongly associated with parent than child-reported scores, while child-reported negative parenting and age were more strongly associated with child than parent-reported scores.

## Discussion

This study used data from a diverse probability-based sample of immigrant children and their caregivers to examine associations between characteristics of neighbourhood and family contexts and children’s mental health. Overall, results showed that negative parenting behaviour was consistently associated with higher levels of parent- and child-reported internalizing and externalizing problems, while positive parenting behaviour was associated with lower levels. In addition, neighbourhood social disorder, parental trauma exposure, and parental distress were associated with higher levels of parent-reported internalizing and externalizing problems, while neighbourhood immigrant concentration were associated with fewer parent-reported externalizing problems. Apart from parenting behaviour, there were few significant correlates of child-reported mental health with only male gender and age being associated with greater internalizing and externalizing problems respectively. The majority of significant correlates were informant-specific, with only negative parenting demonstrating significant cross-informant associations with externalizing problems.

Results showing the importance of parenting behaviour for immigrant children’s mental health are consistent with the large body of evidence on associations between parenting and common mental disorders among general population and immigrant samples [47, 48]. Inclusion of parental distress and parenting behaviours reduced the associations of more distal family and neighbourhood level correlates, including parental trauma exposure and neighbourhood social disorder. While the cross-sectional nature of the data precludes formal testing of mediation effects, our results are consistent with the Family Stress Model and growing evidence of pathways between socio-economic stressors, parental distress, harsh parenting, and poor mental health outcomes among immigrant and refugee children [49, 50]. Associations between parental trauma exposure and distress and parent reports of child mental health problems remained significant after the inclusion of parenting variables in the model, which suggests other potential mechanisms beyond the broad dimensions of positive and negative parenting assessed in this study. Respondent bias may also partially account for these informant-specific associations, given evidence that parents who have experienced trauma and distress may report more negatively on their children’s mental health [51].

As in previous research [4], higher neighbourhood-level immigrant concentration was consistently associated with fewer parent-reported externalizing problems. It has been hypothesized that living in a neighbourhood with immigrants of similar socioeconomic, ethno-cultural, or migration backgrounds may confer a protective effect by enhancing social support and collective efficacy [17]. On the other hand, family socio-demographic characteristics widely assumed to be risk factors for children’s poor mental health such as household poverty and refugee background were not found to be significant correlates in this study. Extant research on mental health risk associated with refugee status is mixed, with some studies showing comparable levels of mental health between refugee children and the general population while others indicating elevated risk among refugees [15, 52]. Given significant heterogeneity in exposures to mental health stressors among immigrant and refugee groups, adverse migration experiences may be a more sensitive risk factor for poor outcomes than broad categorisations of immigrant versus refugee status. Notably, a representative survey with adult refugees and immigrants in the United States found that pre- and post-migration trauma was associated with mental distress and illness across both groups irrespective of immigrant class [7].

Consistent with previous research [53], household low-income status was not a significant correlate of mental health despite immigrant children being significantly more likely to live in poverty. This paradox may be explained by immigrants’ social and cultural resources such as the retention of cultural values and practices that protect against the adverse effects of poverty [54]. In the current study, stratified sampling methods to increase representation of immigrant children resulted in a sample that was skewed towards a restricted and lower range of household income, which may also contribute to our present findings.

Finally, the predominantly informant-specific associations found in this study as well as the few significant correlates of child self-reported mental health merit further investigation with immigrant samples. Previous research suggests that parent–child discrepancies in mental health ratings and their associations with neighbourhood and family correlates may be more pronounced among immigrant families due to divergent cultural expectations about parenting and child adjustment [29]. Substantial inter-informant variation in associations of almost all mental health correlates highlights the risk of relying on single informant assessments among immigrant samples. Future research with immigrant and refugee groups, particularly research aimed at identifying targets for intervention, should devote greater attention to the collection, interpretation, and integration of multi-informant data to avoid misidentification of mental health correlates in this population.

### Strengths and limitations

Strengths of the study include the use of a probabilistic, ethnically and linguistically diverse community sample of immigrant children and inclusion of a broad spectrum of migration-specific and universal mental health correlates in the neighbourhood and family contexts. Multi-informant data enabled systematic testing of the extent to which mental health correlates differed between parents and children. Study limitations include the cross-sectional nature of the study design, which precludes causal inferences and formal testing for mediation effects. Skewness of several study variables (e.g., parental English fluency) may have reduced the reliability of certain estimates, as shown by their larger standard errors. While many of the study measures have been widely used with diverse ethnocultural groups, there is limited evidence on their psychometric properties in immigrant and refugee samples specifically [55]. A validity study of the CBCL/YSR with Somali refugees, for instance, found excellent internal consistency reliability but poor criterion validity for the YSR, highlighting the need for rigorous psychometric evaluation of standardized instruments when administered to diverse immigrant and refugee groups [56]. Future studies on immigrant child and youth mental health would benefit from further investigation of the cross-cultural validity of these measures, while also addressing potential method effects (e.g., positively worded versus negatively worded items) and measurement error through structural equation modelling methods. Finally, while concept coverage was broad within the neighbourhood and family contexts, our models did not include other important contextual factors that influence child mental health such as school environment and peer relationships.

## Conclusions

This study shows that parenting behaviour, parental distress, and parental exposure to trauma are important correlates of immigrant children’s mental health. While not unique to immigrants, the primacy of these processes for immigrant children and families warrants particular attention given the heightened risk of exposure to migration-related adverse experiences that threaten parental and family well-being. To prevent or mitigate cascading effects on child mental health, it is imperative to invest in developing and testing trauma-informed and culturally responsive mental health and parenting interventions for immigrant families [57]. Triangulation of information from multiple informants is crucial when conducting research to identify targets for intervention, as significant variation in parent and child-reported correlates may result in misidentification of neighbourhood and family factors that matter most for immigrant children’s mental health.

## Data Availability

The data that support the findings of this study are not publicly available due to inclusion of information that could compromise participant privacy. The variance–covariance matrix and code used in the study can be made available upon request for research purposes only.
